# The potential of sedimentary ancient DNA for reconstructing past sea ice evolution

**DOI:** 10.1038/s41396-019-0457-1

**Published:** 2019-06-24

**Authors:** Stijn De Schepper, Jessica L. Ray, Katrine Sandnes Skaar, Henrik Sadatzki, Umer Z. Ijaz, Ruediger Stein, Aud Larsen

**Affiliations:** 1 NORCE Climate, NORCE Norwegian Research Centre AS, Bjerknes Centre for Climate Research, Jahnebakken 5, 5007 Bergen, Norway; 2 NORCE Environment, NORCE Norwegian Research Centre AS, Nygårdsgaten 112, 5008 Bergen, Norway; 3 Department of Earth Science, University of Bergen, Bjerknes Centre for Climate Research, Jahnebakken 5, 5007 Bergen, Norway; 4 University of Glasgow, School of Engineering, Oakfield Avenue, Glasgow, G12 8LT UK; 5 Alfred Wegener Institute Helmholtz Centre for Polar and Marine Research, Am Alten Hafen 26, 27568 Bremerhaven, Germany; 6 MARUM and Faculty of Geosciences, University of Bremen, P.O. Box 330440, 28334 Bremen, Germany; 7 Present Address: Research School of Earth Sciences, Australian National University, Canberra, ACT 2601 Australia

**Keywords:** Climate change, Molecular ecology, Climate-change ecology, Next-generation sequencing

## Abstract

Sea ice is a crucial component of the Arctic climate system, yet the tools to document the evolution of sea ice conditions on historical and geological time scales are few and have limitations. Such records are essential for documenting and understanding the natural variations in Arctic sea ice extent. Here we explore sedimentary ancient DNA (aDNA), as a novel tool that unlocks and exploits the genetic (eukaryote) biodiversity preserved in marine sediments specifically for past sea ice reconstructions. Although use of sedimentary aDNA in paleoceanographic and paleoclimatic studies is still in its infancy, we use here metabarcoding and single-species quantitative DNA detection methods to document the sea ice conditions in a Greenland Sea marine sediment core. Metabarcoding has allowed identifying biodiversity changes in the geological record back to almost ~100,000 years ago that were related to changing sea ice conditions. Detailed bioinformatic analyses on the metabarcoding data revealed several sea-ice-associated taxa, most of which previously unknown from the fossil record. Finally, we quantitatively traced one known sea ice dinoflagellate in the sediment core. We show that aDNA can be recovered from deep-ocean sediments with generally oxic bottom waters and that past sea ice conditions can be documented beyond instrumental time scales. Our results corroborate sea ice reconstructions made by traditional tools, and thus demonstrate the potential of sedimentary aDNA, focusing primarily on microbial eukaryotes, as a new tool to better understand sea ice evolution in the climate system.

## Introduction

Arctic sea ice is a crucial component of the Arctic climate system, but it is probably one of the least well-documented and understood components, especially on historical and geological timescales. A major reason is that satellite records only cover the past decades of Arctic sea ice evolution, providing a (too) short account of sea ice variability during a time when anthropogenic greenhouse gas emissions were already rising. To grasp natural Arctic sea ice variability, it is essential to generate sea ice records beyond the observational and historical records via sources of climate information from natural archives (proxies). Sea ice proxies are still under development (e.g., [1, 2]) and mostly utilize chemical signatures (the biomarker IP_25_) or microfossil assemblages (diatoms, dinoflagellate cysts) from phytoplankton associated with sea ice to reconstruct the past Arctic sea ice cover. While these methods have advantages such as availability of large datasets, rapid measurement, seasonal sea ice reconstructions, potential for quantitative reconstructions, they do have some immanent limitations such as poor preservation, indirect relation with sea ice, absence under permanent sea ice or a limited regional application [3, 4].

Here we demonstrate the potential of sedimentary aDNA metabarcoding for sea ice reconstructions and of specific sea ice organisms as palaeo-sea ice indicators, focusing mainly on DNA originating from the microbial eukaryotes. Compared to the traditional proxies, our approach has a strong advantage through a more direct link to sea ice via the broader eukaryote (sea ice) community and/or individual sea ice organisms, whose genetic signatures (environmental DNA) have been preserved in sediments and can be used to characterize past biodiversity [5]. Molecular techniques employed on sea ice communities itself have previously documented characteristic and unique DNA signatures in open-ocean, seasonal and permanent sea ice environments [6, 7]. DNA signatures from surface ocean microorganisms have been detected in marine surface sediments, revealing diversity beyond the fossil record [8, 9]. Moreover, aDNA has been documented from Late Quaternary sediments (e.g., [10, 11, 12]) and building on early studies in Antarctica [13], we explore here for the first time its potential for reconstructing Arctic sea ice conditions in the Late Quaternary. Finally, DNA sequencing can be done at competitive speed, cost and ease of use due to on-going technological advances [14].

## Materials and methods

### Sediment cores and samples

**Fig. 1:**
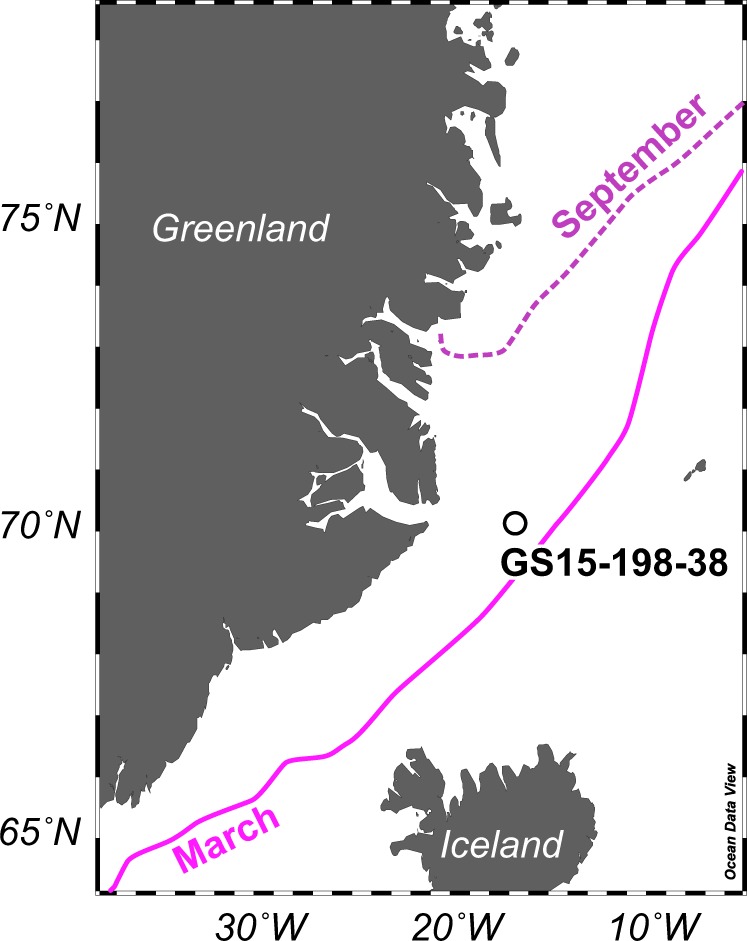
Map of the East Greenland Sea and the Station GS15-198-38 with the median September and March sea ice extent (1981–2010) [72]

A multicore (MC) and a 19.6-m long Calypso core (CC) were recovered from the East Greenland Sea (Station GS15-198-38; 70˚07.612′ N, 17˚39.765′ W; 1610 m water depth; Fig. 1) in the summer of 2015 during the Ice2Ice cruise with the RV G.O. Sars. Both cores were split on the ship and sampled immediately using sterile 20 mL polypropylene syringes. Eight sediment samples were taken at random depths in undisturbed intervals of the Calypso core, and the sediment interval 0–1 cm was collected from the multicore. Syringes filled with sediment were put into individual plastic bags and frozen immediately at −80 °C until molecular analyses. The detailed sampling method for molecular analyses is presented in the Suppl. Information. The sediment cores were placed in cool storage (4 °C) and sampled post-cruise for organic biomarker analyses and palynology. Total organic carbon and biomarker analyses were performed at the Alfred Wegener Institute (Bremerhaven, Germany) following techniques described in refs. [15, 16]. Semi-quantitative sea ice estimates based on the phytoplankton-IP_25_ (PIP_25_) index were calculated following ref. [1]. Palynological laboratory procedures were performed at Palynological Laboratory Services Ltd. (Holyhead, UK) using a standard procedure [17]. More details on the biomarker and palynology laboratory protocols are given in the Suppl. Information. All analyses were done on samples collected at the same sampling depth.

The age model for the core is based on linear interpolation between 10 tie points determined via AMS ^14^C dating down to 345 cm (45,128 cal yr BP) and a 5-cm resolution *N. pachyderma* sinistral isotope stratigraphy [18] from 345 to 660 cm (marine isotope stage 5e, ~123,000 years ago). Full details of the age model and tie points can also be found in the Suppl. Information.

### Metabarcoding, bioinformatics and droplet digital PCR

Full details about the methods, protocols and bioinformatical pipelines are available in the Suppl. Information. In brief, sediment subsampling, DNA purification and PCR set-up were all conducted in access-restricted, purposed rooms at the Norwegian Research Centre (NORCE, Bergen) with protective lab wear and clean equipment in order to minimize cross-contamination risk and sample contamination with modern DNA in compliance with recommendations [19]. Our metabarcoding strategy targeted a broad diversity of eukaryotic organisms through amplification of the V7 hypervariable region of the small subunit ribosomal RNA (SSU rRNA) gene [20]. Briefly, 0.5 pmol of each primer 1183mod (5′-AATTTGACTCAACRCGGG-3′) and R1443mod (5′-GRGCATCACAGACCTG-3′) [20, 21] was added to 50 µl PCR reactions containing 5 µl aDNA as template, 5 µg molecular biology grade Bovine Serum Albumin (New England Biolabs, Ipswich, MA, USA), 5 µmol of each dNTP, 0.2 U Phusion high-fidelity DNA polymerase (New England Biolabs, Ipswich, MA, USA), 1X buffer and ultrapure water [20]. Metabarcoding libraries were prepared in three steps: (1) 30 cycles of amplification of eukaryote SSU V7 target fragments from aDNA samples, (2) adapter-ligation PCR (10 cycles) to append Illumina adapter sequences to amplicons from Step 1, and (3) barcode-ligation PCR (15 cycles) to append forward and reverse Illumina barcodes (8 nt) to amplicons from Step 2 (details in Suppl. Information). All PCR products were twice-purified using magnetic beads (MagBio, Gaithersburg, ME, USA) at a PCR product:bead volumetric ratio of 1:1.8 in the first round, and 1:1 in the second round. Dual-indexed amplicon libraries were pooled in equimolar ratios, then the pooled library was purified with magnetic beads at bead volume ratio of 1.0 to ensure complete removal of primer dimers. Sequencing on an Illumina MiSeq platform using v.3 chemistry and 600 cycles (300 bp × 2) was conducted at the Norwegian High-Throughput Sequencing Centre in Oslo, Norway.

The raw data were quality-trimmed and error-corrected using published bioinformatics tools (Suppl. Information). This was followed by pooling, dereplication, sorting, singleton removal, operational taxonomic unit (OTU) clustering using VSEARCH v.2.8.1 [22] at 97% similarity, de novo chimera removal, and mapping reads back to OTUs. OTUs were taxonomically classified against the Protist Ribosomal Reference database v.4.10.0 (PR2) [23]. In total, 1042 OTUs were generated at the 97% similarity level, of which 65 OTUs were observed in pooled sampling and extraction controls. The OTUs that appeared in sampling and extraction controls samples, to which no sediment or template DNA had been added, were defined as sequence “contaminants” and subsequently informatically excluded from all sediment sample data prior to statistical analysis of the remaining 977 OTUs. All statistical analyses were conducted in the R statistical computing environment [24].

Droplet digital PCR (ddPCR) analysis was conducted to quantify the abundance of the sympagic dinoflagellate *Polarella glacialis* in sedimentary aDNA. Primers amplifying the ribosomal ITS1 region of *P. glacialis*, Polarella-ITS-44F (5′-CGACTGGGTGGAGATGGTTG-3′) and Polarella-ITS-138R (5′-CCCAGGTGTTTAAGCCAGGT-3′), were designed and tested for efficiency and specificity (see Supplementary Material for a detailed protocol description). All clones (*N* = 10) from ddPCR amplification of *P. glacialis* ITS1 from a mixture of all six DNA subsamples from the core surface sediment gave best hit to *P. glacialis* when compared to GenBank using the *blastn* algorithm. PCR reactions were performed in C1000 Touch thermocycler with deep-well module (Bio-Rad). PCR products were cloned using a standard cloning kit and Sanger sequenced for verification purposes. Quantitative amplification of *P. glacialis* ITS1 gene fragments from aDNA was followed by droplet generation and post-PCR enumeration. The ddPCR results were normalised to *P. glacialis* ITS1 copies per g sediment.

## Results

### Palynology

Most samples recorded very few dinoflagellate cysts (concentrations below 50 cysts/g sediment, see Table 1). The surface sample (sample 1 cm) was dominated by cysts of *Protoceratium reticulatum*. *Nematosphaeropsis labyrinthus* and *Impagidinium pallidum* were abundant and the sample also contained heterotrophic taxa (*Brigantedinium*, Round Brown Cysts). The samples at 24 and 249 cm contained a characteristic low-diversity assemblage in higher concentrations (respectively 286 and 187 cysts/g sed). Sample 24 cm recorded an autotrophic dinoflagellate cyst assemblage dominated by *Spiniferites* (including *Spiniferites elongatus*) and the common presence of *Nematosphaeropsis labyrinthus* and cysts of *Protoceratium reticulatum*. Such assemblage is not typically associated with sea ice. In contrast, sample 249 cm was dominated by heterotrophic taxa like *Islandinium minutum*, *Brigantedinium* and Round Brown Cysts. This sample also recorded cysts of the sea ice dinoflagellate *Polarella glacialis* (*n* = 2). Together, the dinoflagellate cyst assemblage indicated a nutrient-rich environment, associated with (seasonal) sea ice. Fresh water algae *Halodinium* and *Pediastrum* were also recorded.

**Table 1:** Selected palynological and organic biomarker data from the studied samples of site GS15-198-38. Ages based on the age model presented in the Suppl. Information. Samples indicated with * are not cal yr BP (calender years before present (1950)), but years according to LR04 stack. MC = Multicore, CC = Calypso Core

Sample (cm)	Core	Depth (cm)	Age (cal yr BP)	Dinocyst concentration (cysts/g sed)	Cyst of *P. glacialis* (n)	TOC (%)	IP_25_ (µg/gTOC)	Brassicasterol (µg/gTOC)	Dinosterol (µg/gTOC)	Campesterol (µg/gTOC)	ß-Sitosterol (µg/gTOC)	HBI-III (triene Z) (µg/gTOC)
001	38MC-B	1	<1,903 AD	1351 ± 169	0	0.621	0.1467	31.296	7.985	27.061	35.313	0.0932
024	38CC	24	17,408	281 ± 43	0	0.338	0	5.790	0.242	1.394	8.750	0
099	38CC	99	21,862	20 ± 8	0	0.358	0	2.490	0.000	0.328	4.870	0
169	38CC	169	26,233	9 ± 9	0	0.249	0.0347	5.198	0.704	1.511	9.702	0
249	38CC	249	33,702	113 ± 17	2	0.381	1.0402	27.458	3.235	6.672	22.574	0.0578
289	38CC	289	38,497	11 ± 5	0	0.272	0.0546	3.178	0.000	0.635	8.328	0
390	38CC	390	51,090*	8 ± 4	0	0.216	0	3.654	0.000	0.393	8.999	0
490	38CC	490	66,595*	48 ± 22	0	0.324	0.0256	7.746	2.369	5.050	11.088	0
590	38CC	590	97,761*	37 ± 14	0	0.272	0	3.804	0.056	0.638	6.763	0

### Biomarkers

**Fig. 2:**
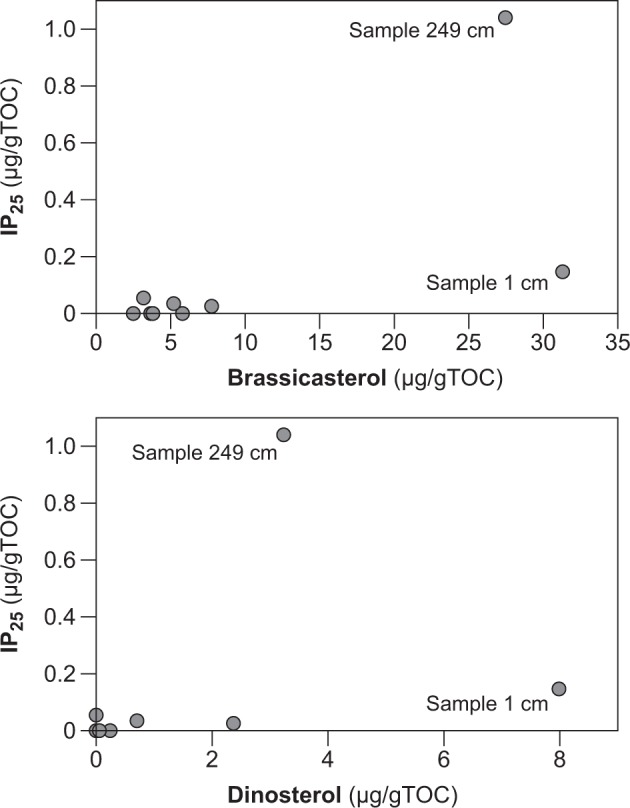
Cross-plot of IP_25_ biomarker vs. brassicasterol and dinosterol

The mono-unsaturated highly branched isoprenoid IP_25_, or “Ice Proxy with 25 carbon atoms”, is produced by certain Arctic sea ice diatoms [25, 26]. In surface sediments of the modern ocean, IP_25_ is most abundant where seasonal sea ice occurs, whereas it is rarely recorded in permanent sea ice and absent in sea-ice free conditions [4, 27]. Its occurrence in sediments provides evidence for past sea ice occurrence. We recorded IP_25_ in the surface sample and four samples of the sediment core. The highest IP_25_ value (1.04 µg/g TOC) was recorded in sample 249 cm, where also high values of HBI-III (0.06 µg/g TOC), brassicasterol (27.46 µg/g TOC) and dinosterol (3.24 µg/g TOC) were recorded (Fig. 2 and Table 1). In the surface sample, high phytoplankton biomarker values were recorded, and also IP_25_ was up to 0.15 µg/g TOC. In the other samples, IP_25_ was zero or maximally 0.05 µg/g TOC, and also brassicasterol (<7.7 µg/g TOC) and dinosterol (<2.37 µg/g TOC) showed low values. IP_25_ only indicates presence or absence of seasonal sea ice, but can be used in combination with phytoplankton biomarkers (e.g., dinosterol or brassicasterol) to calculate the PIP_25_ index, which allows to reconstruct sea ice and sea surface conditions, respectively [1]. Based on the individual biomarker data and the P_B_IP_25_ and P_D_IP_25_ indices (0.65–0.66), our surface sample and sample 249 cm indicate seasonal sea ice conditions. In all samples where IP_25_ is (near) zero, this can be interpreted as either sea ice free or permanent sea ice conditions due to limitations of the PIP index [1, 25, 27]. Also in those samples, near zero values of brassicasterol and dinosterol indicate limited phytoplankton productivity and permanent sea ice conditions, rather than sea ice free conditions where high phytoplankton productivity is expected. The β-sitosterol and campesterol biomarkers are abundant in sample 249 cm (22.57 µg/g TOC and 6.67 µg/g TOC, respectively), and show generally low values in the other samples (<11.09 µg/g TOC and <5.05 µg/g TOC, respectively), except for the surface sample.

### Metabarcoding

**Fig. 3:**
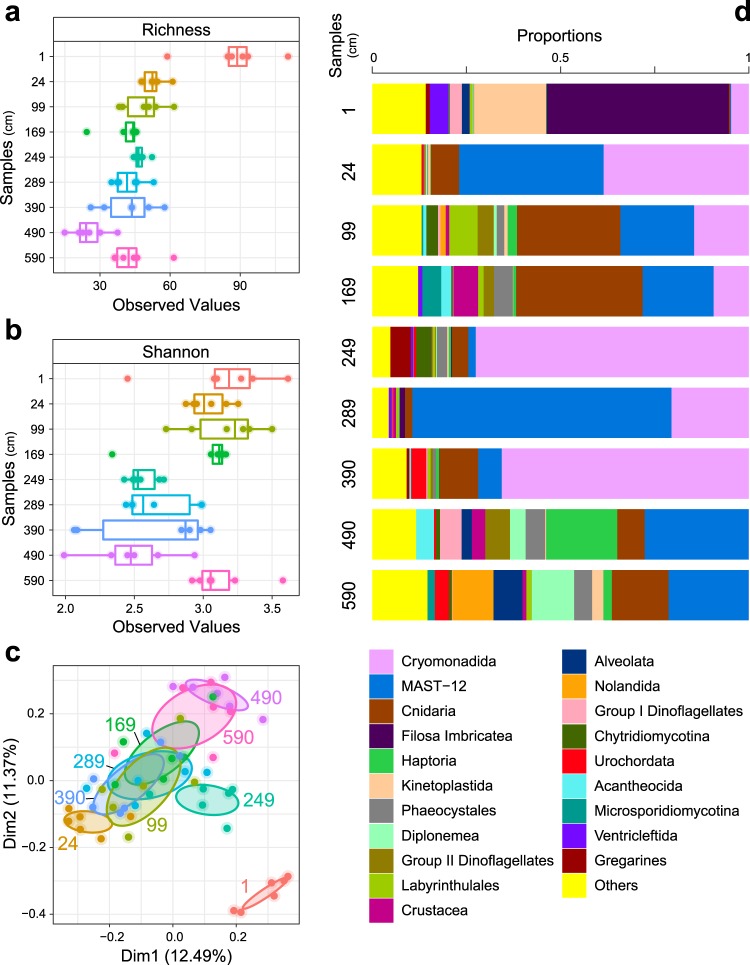
Diversity analysis of metabarcoding libraries amplified from one surface sample and eight downcore samples at station GS15-198-38, East Greenland Sea. **a** Boxplot showing predicted OTU richness. **b** Boxplot showing Shannon index values. **c** Principle coordinates analysis (PCoA) of unweighted UniFrac dissimilarity. Coloured labels refer to sample depths. **d** Pooled (*N* = 6) relative abundances of family-level taxonomic identification of OTUs for each sediment sample. Composite bars show the 20 OTUs with highest relative abundance, and all remaining OTUs are collectively shown as “Others”. Best-hit classifications were performed by querying the Protist Ribosomal Reference (PR2) database v.4.10.0 with metabarcodes using the blast algorithm. Sample IDs (y-axis) show core depth in cm and taxon bar widths (“Proportions” on the x-axis) indicate relative abundance (%) of taxonomic groups in each sediment sample

DNA yield from 6 subsamples for each of 9 sediment samples (54 measurements in total) varied from undetectable (limit of detection 200 pg per assayed sample volume) to 1795 ng DNA per g sediment, with a rapid drop in recoverable double-stranded DNA (dsDNA) from surface to downcore sediments. After sequencing the 18S rDNA gene, quality-filtering, merging, clustering with singleton removal, and *de novo* chimera removal resulted in 143,750 reads that clustered into 977 aDNA operational taxonomic units (OTUs) with a 97% similarity cut-off. See Suppl. Information and Suppl. Table 1 for details on sequence data metrics. The 977 OTUs were used to characterize the genetic diversity revealed by aDNA metabarcoding analysis as α- and β-diversity. The predicted genetic diversity *within* each sediment layer, or α–diversity [28], was calculated using two standard ecological diversity measures, the rarefied genetic richness (Fig. 3a) and the Shannon index (Fig. 3b). The rarefied richness was distinctly higher in the surface sample compared to the downcore samples. This difference was not preserved in the Shannon index, which considers both OTU richness and relative abundance within a sample. In the downcore samples, rarefied richness varied from approximately 30 to 60 OTUs per subsampling iteration (Fig. 3a). Pairwise analysis of variance (ANOVA) tests on rarefied richness estimates indicated significant differences between the genetic diversity present in the different samples (Suppl. Table 2). The β-diversity, or genetic diversity *between* sediment samples, is represented as the unique fraction distance (UniFrac, (ref. 29)). Principle coordinates analysis (PCoA) of the unweighted UniFrac distance matrix demonstrates distinct clustering of some samples, while others overlap (Fig. 3c). Most notably, the surface sample genetic diversity was highly distinct from the downcore samples. Among the downcore samples, some degree of distinction between sample clusters exists, with the strongest genetic dissimilarity between the samples 24, 249 and 490 cm (Fig. 3c).

Constrained correspondence analysis (CCA) of metabarcoding results (OTUs) using dinoflagellate cyst and biomarker concentrations could explain only 14.4% of the observed genetic diversity in the metabarcoding results (Total inertia 9.9527, Constrained inertia 1.4305, Unconstrained inertia 8.5219) (Suppl. Fig. S1). This indicates that the indicator measures have poor discriminatory power for the observed genetic diversity. Unconstrained PCoA analysis revealed that the strongest genetic distinction was observed between the surface and downcore samples (Fig. 3c), in part, due to higher dinocyst abundances and concentrations of dinosterol and brassicasterol in the surface sample.

Examination of the taxonomic diversity captured by metabarcoding analysis of aDNA revealed a rich taxonomic coverage, with representation of protists, fungi, diatoms, as well as invertebrate and vertebrate metazoans (Fig. 3d). Of the 977 OTUs generated from the metabarcoding results, 158 (16% of OTUs) were not classified at any taxonomic level. The OTUs with highest relative abundance in the aDNA metabarcoding data had highest sequence similarity to Cerocozoans (Stramenopiles: Rhizaria) (116 OTUs, 34.8% of all reads, 0–76% per sample) and two marine stramenopile (MAST) clades [30] (54 OTUs, 27.7% of all reads, 0.3–68% per sediment layer). Metazoans were also present in high relative abundance (127 OTUs, 14.8% of all reads, 2–41.6% per sediment layer) in several samples and were represented by sequences with highest similarity to reference sequences from arthropods, flatworms, cnidarians, tunicates, hydrozoans and annelids. Diatoms comprised only 0.1% of all reads and represented 0–2% of reads per sediment layer (11 OTUs), with reads resembling both centric and pennate diatoms. Dinoflagellate-like reads comprised 2% of total sequence reads (56 OTUs, 0.2–13% of reads per sample) including reference sequences from Syndiniales (mainly), *Protoperidinium*, Suessiales and *Gymnodinium*. A complete table of OTUs with taxonomic classifications is provided in the Suppl. Table 3.

Next, we linked individual OTUs to environmental variables, measured on the same samples (i.e., from the same sample depth). The environmental variables employed were concentrations of dinocysts (measure for productivity), brassicasterol and dinosterol (phytoplankton biomarkers, productivity), and the sea ice diatom biomarker IP_25_, and its derived indices P_B_IP_25_ and P_D_IP_25_. We used sparse partial least squares discriminant analysis (sPLS-DA) on the downcore samples and identified 348 OTUs with significant discriminatory power (Suppl. Fig. S2). Pairwise correlation analysis of discriminatory OTUs against the measured environmental parameters revealed significant (Adj. P < 0.05) positive correlations (Kendall’s *tau* 0.430–0.498) with IP_25_ and P_B_IP_25_ for four putative cercozoan OTUs (OTU_348, OTU_4579, OTU_4620, OTU_4660), one OTU resembling a polar centric diatom (OTU_5051) and a *Gymnodinium*-like OTU (OTU_333) (Suppl. Table 4).

### Droplet digital PCR of the dinoflagellate Polarella glacialis

**Fig. 4:**
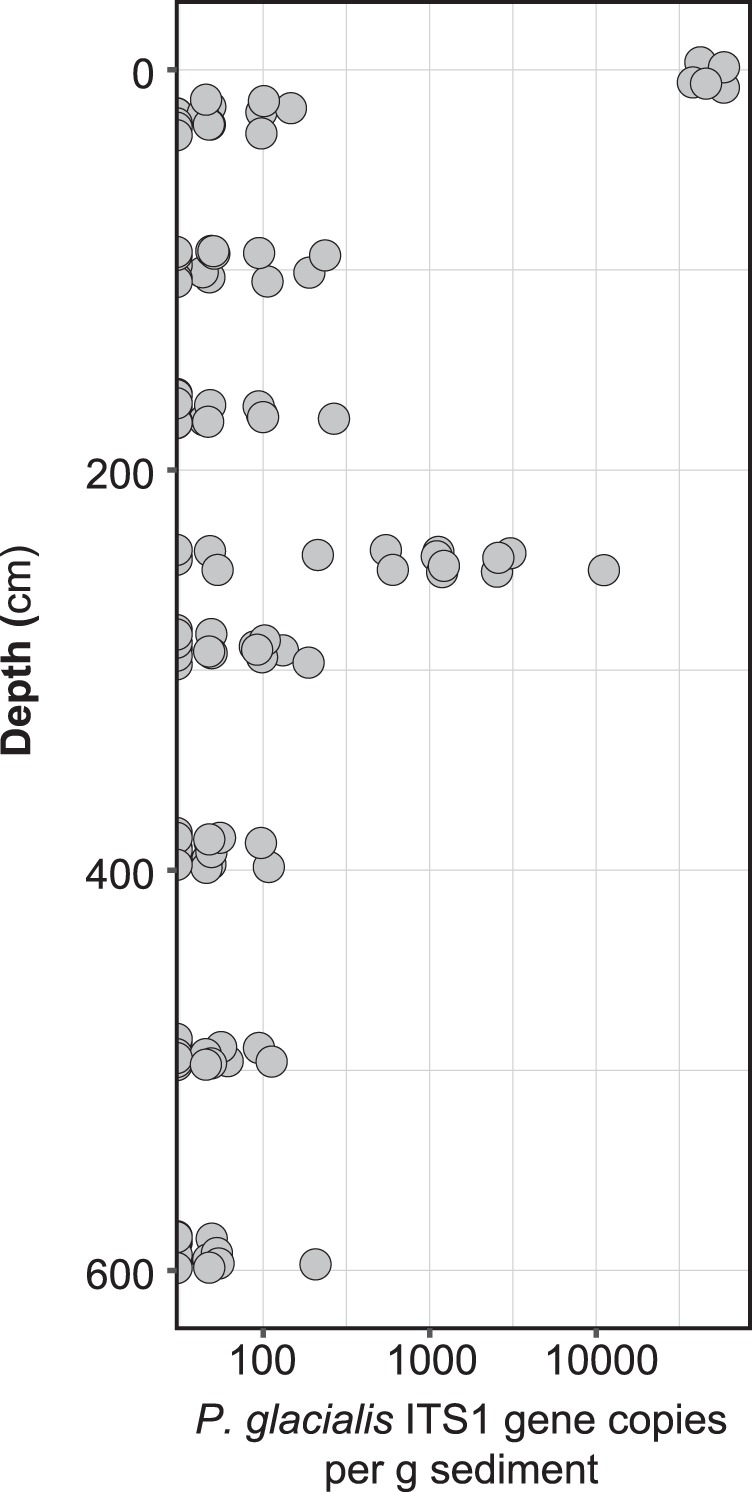
Droplet digital PCR (ddPCR) quantification of *P. glacialis* ITS1 gene copies (note logarithmic *x*-axis) as a function of depth

In addition to qualitative investigation of sedimentary aDNA using metabarcoding, we employed a quantitative approach (droplet digital PCR, or ddPCR) to specifically quantify DNA sequences from *Polarella glacialis*. We chose to target this species because it is a known sea-ice associated dinoflagellate that was identified during the microscope analysis. Using PCR primers designed to specifically amplify the *P. glacialis* ribosomal RNA ITS1 region (Suppl. Information), we observed patchy distribution of *P. glacialis* DNA in the different samples as well as within replicates of the same sample, with gene copy abundances ranging from 0 to 58 533 gene copies g/sediment (Fig. 4). Highest *P. glacialis* ITS1 gene copy abundances were observed in the surface sediment layer. Interestingly, several subsamples from 249 cm depth (33,678 cal yr BP) contained an approximately 10- to 100-fold higher abundance of detectable *P. glacialis* ITS1 gene copies compared to the other downcore samples (<40 to 266 copies g/sediment).

## Discussion

In our attempt to explore the applicability of using sedimentary aDNA to reconstruct Arctic sea ice on Late Quaternary time scales we demonstrate that DNA from Arctic sediments of ca. 100,000 years old is well preserved, even from a region with generally oxic bottom waters, and that it can be used to describe the sea ice history. We recorded aDNA in all samples of our sediment core in the Greenland Sea (Fig. 3d). Our lowermost sample 590 cm, dated to almost ~100,000 years ago, currently provides the oldest record of sedimentary aDNA in the Arctic. Of the 977 OTUs detected by metabarcoding analysis of aDNA, 230 were present in this sample, the majority of which were classified as Euglenozoa, Stramenopiles (MAST), Cnidaria, Fungi and Amoebozoa. Deep-ocean sediments provide a stable, low-temperature environment that may aid the preservation of DNA in marine anoxic and oxic, subsurface settings (e.g., [9, 12, 13, 31–34]) underpinning that sedimentary aDNA can indeed become a useful additional proxy to bolster our understanding of Arctic and oceanic change in the Late Quaternary, possibly even beyond ~100,000 years [32, 35].

### Metabarcoding reveals changes in past (sea ice) environments

With our generalist approach, using a moderately short fragment of ~260 base pairs targeting a wide diversity of eukaryotic organisms, of which we focus specifically on the micro-sized ones that compare best with traditional sea ice proxies, we gathered the broad molecular signature of Late Quaternary sediments in the East Greenland Sea (Fig. 1). The considerably higher diversity and unique metabarcode signature in the surface sample (sample 1 cm) compared to the downcore samples (Fig. 3a, c) can be attributed to better preservation in the surface sample, which reflects modern conditions. A higher degradation of the DNA signal is to be expected with increasing age [36, 37] and possibly affects the metabarcoding results in the two oldest downcore samples.

In the downcore samples (24 to 590 cm), the metabarcoding results show a remarkably strong agreement with the pattern derived from traditional sea ice proxies (palynology and biomarkers). In samples 24 to 390 cm, a consistent metabarcoding signature with abundant marine stramenopile and Cercozoa sequences occur (Fig. 3c, d). The marine stramenopile clade MAST-12 is a cosmopolitan group of heterotrophic flagellates occurring in planktic settings and sediments of both oxic and anoxic marine and fresh water environments [38, 39]. A link between the diverse MAST-12 group and sea ice is currently not documented in the modern ocean. In contrast, Cercozoa are important heterotrophic protists occurring in a multitude of marine environments, including open water, marine sediments and sea ice [7, 39–42]. The *Cryothecomonas* lineage of the Cryomonadida consists of heterotrophic grazers that forage on sea-ice brine communities [43]. The most abundant OTUs in the metabarcoding dataset most closely resemble reference sequences from this group of sea-ice associated protists in all samples younger than ~51 kyrs (samples 24 to 390 cm), thus suggesting the presence of sea ice. Their absence from the two eldest samples (~67 and 98 kyr) could be a true signal, but also a preservation or detectability artefact. It is important to note that Cercozoans have only been reported in the geological record through the use of molecular techniques [44]. Also worth highlighting, is that during the Last Glacial Maximum (~22 and ~26 cal kyr BP, samples 99 and 169 cm respectively), Cnidarians were conspicuously present in the record (Fig. 3d). Whether there is a link between these organisms and sea ice cover is speculative at this point, but Cnidarians have been observed both in and under sea-ice in the Arctic [45, 46]. In the absence of a sediment metabarcode reference database from sea ice regions, it is difficult to unquestionably assign this signature dominated by marine stramenopiles and Cercozoa sequences to sea ice. However, the absence of the sea ice biomarker IP_25_ and low dinoflagellate cyst concentrations (low productivity) likely reflects a permanent sea ice cover between ~17.5 (24 cm) to 51 cal kyr BP (390 cm), except at ~33.7 cal ka BP (sample 249 cm, see below). IP_25_ is usually absent in sediments underlying open water and under permanent sea ice conditions, but the low concentration of phytoplankton productivity biomarkers (brassicasterol, dinosterol) and dinoflagellate cysts indicate a limited productivity that is most consistent with a permanent or extended sea ice cover. The metabarcode signature between ~17.5 to 51 cal kyr BP may thus be reflecting a permanent sea ice cover. The elevated dinocyst concentrations of mainly *Spiniferites* at 17.5 cal kyr BP suggests productivity, possibly related to a return to seasonally sea ice free conditions in the region.

**Fig. 5:**
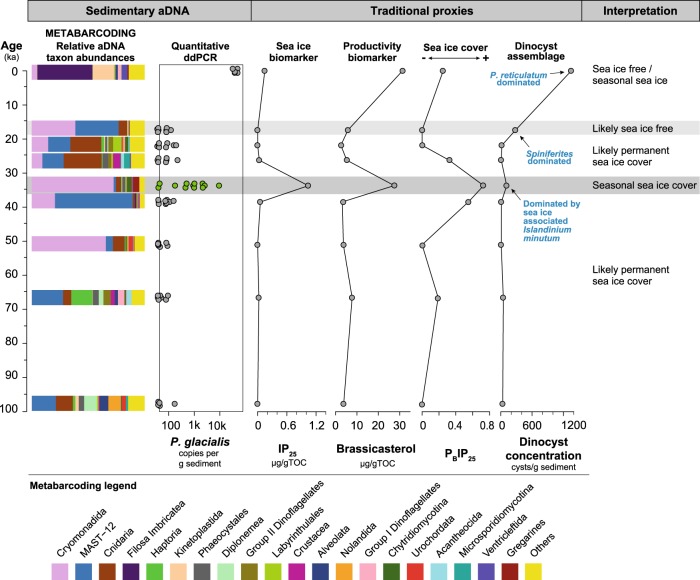
Comparison of our novel sedimentary aDNA approach (metabarcoding and ddPCR) with traditional proxies (biomarkers and palynology) for sea ice reconstructions over the last ~100,000 years at Site GS15-198-38 in the Greenland Sea

Interestingly, the PCoA analysis identifies sample 249 cm (~33.7 cal ka BP) to have a different signature compared to the other samples (Fig. 3c), indicating a biodiversity and environmental shift. This shift is best explained by the disappearance of permanent sea ice and shift towards a seasonal sea ice cover. We record a higher value of IP_25_, while also brassicasterol, dinosterol and dinocyst concentrations (productivity) increased. The dominance of the sea ice associated dinoflagellate *Islandinium minutum* and presence of the sea-ice dinoflagellate *Polarella glacialis*, both detected using microscopy (cysts) and genetic tools (see below) indicate seasonal sea ice in this sample (Figs. 4, 5). A shift from permanent to seasonal sea ice in the Greenland Sea implies a substantial retreat of the sea ice edge, likely associated with Arctic climate warming around that time. Although speculative without a more detailed record, the timing of the shift in our record around 33.7 cal kyr BP corresponds favorably to Greenland Interstadial 6 (33,690–33,310 cal yr BP, Rasmussen et al. 2014), when the (eastern) Nordic Seas were largely sea ice free and Greenland temporarily warmed [16, 47, 48].

### Exploring individual genetic sequences as sea ice indicators

A detailed correlation analysis of the aDNA metabarcodes and the traditional proxies has identified several potential sea-ice indicator taxa in the geological record. In comparing the genetic data with the IP_25_ biomarker (and its derived indices P_B_IP_25_), we identified significant correlations (Suppl. Fig. S3, Suppl. Table 3) between the biomarker and OTUs belonging to the Cercozoan clades Filosa-Thecofilosea [49, 50], including one *Cryothecomonas*-like OTU [43], and the silicoflagellate clade Filosa-Imbricatea, both of which are common in polar marine environments. Dinoflagellate and diatom sequences are well-known in sea ice [6, 7], but only constitute a minor fraction of the metabarcodes generated from our sedimentary aDNA samples (Suppl. Table 4). The reasons for this underrepresentation relative to other studies (e.g., [36]) are unclear, although differential preservation (e.g., [13, 51, 52]) and/or predation [53] may be possible explanations. Nevertheless, one diatom-like OTU classified as a polar centric mediophyceae (OTU_5051) and one *Gymnodinium*-like OTU (OTU_333) were found to be significantly correlated to measured concentrations of IP_25_. These latter two are discriminatory OTUs for the sample 249 cm, in which the IP_25_ concentration was highest. OTUs classified as known sea-ice associated taxa, such as dinoflagellates in the Suessiaceae family (e.g., *P. glacialis*), were also positively associated with IP_25_, however this association was not found to be statistically significant (Suppl. Fig. S3). Interestingly, we recorded the presence of the sea ice dinoflagellate *P. glacialis* in sample 249 cm (~33.7 cal kyr BP; Fig. 5) using a palynological preparation using cold acids only and sieving at 10 µm. This record represents the oldest fossil record of this species in the Arctic, since cysts of *P. glacialis* were previously only recovered from Arctic [54, 55] and Antarctic [56] surface sediments. It is likely that this small cyst (12–17 µm long and 8–15 µm wide; (ref. 57)) has been misidentified as an acritarch or overlooked in previous palynological studies. Cyst recovery can further be hampered by poor cyst preservation during sedimentation [13], unfavorable preparation techniques and/or low preservation potential (i.e., warm acids, sieving at >10 µm, acetolysis; (ref. 57)).

Because of our metabarcoding data, the detection of *P. glacialis* cysts in our slides, and previous successful identification of *P. glacialis* in paleoenvironmental genomics work in Antarctica [13], we designed a primer amplifying the ITS1 region to identify and quantify (ddPCR) this species in our downcore record. The ddPCR recorded *P. glacialis* in all samples and demonstrated increased abundances in the surface sample (sample 1 cm) and sample 249 cm (Fig. 4). The detection of *P. glacialis* in the surface sample indicates that in modern times, sea ice influenced the coring site. Indeed, the site falls within the mean winter sea ice extent of the satellite era (1980–2010) (Fig. 1). The peak ddPCR value in sample 249 cm occurs together with the major shift in metabarcode signature (discussed above), the record of cysts of *P. glacialis*, and increased IP_25_ concentrations (Fig. 5). Although we have not assessed the relative degradation state of *P. glacialis* target gene fragments in the different samples, the clear peak in *P. glacialis* ITS gene copy numbers is conspicuous and together with the presence of cysts of *P. glacialis* provides strong evidence for seasonal sea ice [55]. This suggests that individual micro-sized sea ice taxa can be targeted and employed for sea ice reconstructions in the Late Quaternary, even when their fossil remains are not or rarely detected using traditional microscopy.

### Challenges with using sedimentary aDNA as a sea ice proxy

We employed a broad taxonomic characterization of the sedimentary aDNA to reconstruct sea ice in the geological past. To achieve the necessary balance between high phylogenetic resolution and DNA detection in geological samples dating back ~100,000 years, we chose a moderate target amplicon length (~260 base pairs). One of the key challenges of a broad sedimentary aDNA metabarcoding detection approach is that the generated sequence information represents an amalgamation of taxa of diverse origins, not only from sea ice. DNA present in marine sediments can reflect biological diversity present in the sediment biome [58, 59], or it may originate from the overlying water column [60, 61] including from ecologically distinct ocean surface biomes such as sea ice [7]. Identifying sea-ice relevant genetic signatures among the genetically diverse signals is a real challenge as all may potentially become incorporated into the extractable and amplifiable sedimentary aDNA pool. The extremely high variation in the number of SSU rRNA (18S) gene copies per cell for different protists (e.g., for ciliates [62]) poses an additional challenge in identifying quantitative trends in biodiversity dynamics [63] in the context of specific climate events. Nevertheless, we were able to identify major biodiversity shifts related to changed sea ice conditions using our metabarcode approach. This may imply that the genetic signature of the surface waters and sea ice environments is in fact adequately captured in the sediment and identified by the metabarcoding. Alternatively, it could also be a reflection of the translative effect on biodiversity in the water column and at the seafloor through benthic-pelagic coupling [64]. Another persistent challenge in molecular ecology, is the common inability to assign organism identity and ecology to gene sequences [65, 66]. This challenge has become pervasive due to the increase in molecular environmental research while functional studies on isolated organisms remain scarce [65, 67]. Although we were able to identify several sea ice related OTUs, originating primarily from the microbial eukaryotic fraction, studies focusing on linking gene sequences to sea ice organisms would generate a larger group of sea ice reference sequences that allow a more detailed understanding and reconstruction of past sea ice change. Targeted molecular approaches for specific taxa, for example ddPCR quantification of *P. glacialis* in this study, partially circumvent these challenges by direct quantitative comparison of taxa abundance with key environmental parameters. In addition, such approach can detect rare taxa whose genetic signal may be masked by the abundant majority [68]. For this reason, we designed shorter primers to quantify the DNA copies of the sea ice dinoflagellate *P. glacialis* in our samples. Shorter (<100 base pairs) fragments are preferential for targeted or quantitative studies [12, 69], particularly from samples in which extensive DNA degradation is expected [70].

In addition to challenges of sourcing detectable DNA signal, contamination with modern DNA may mask the ancient DNA signal. Due to the highly degraded nature of ancient DNA, calls for standardization of protocols enforce strict guidelines for aDNA analysis and data interpretation [71]. In this study, all recommended precautions for protecting sedimentary aDNA samples from modern contamination were strictly adhered to (e.g., [12, 13, 31]). Extensive blank controls for sediment sampling (*N* = 8) and for DNA extraction (*N* = 10) were routinely checked for contamination, and where PCR products were visible on an agarose gel (*N* = 2), purified and sequenced as separate samples. These sequences were bioinformatically removed from all sample data prior to statistical analysis (Suppl. Information). The remaining biodiversity revealed by our metabarcoding approach makes biological sense, with a high diversity of marine-associated taxa. Deeper investigation into DNA degradation state and gene copy numbers would be desirable for drawing stronger conclusions about taxon abundances and genetic variability based on the metabarcoding results alone. Full characterization of sedimentary aDNA pools present in the sediments examined, however, falls outside the scope of this study.

In summary, we have shown how past microbial ecology can be used in climate research by demonstrating that universal metabarcoding and single-species quantitative DNA approaches can characterize sea ice evolution on a Late Quaternary timescale and both corroborate and complement sea ice reconstructions using traditional paleo-sea ice proxies. The major shifts in the Greenland Sea sedimentary aDNA profiles determined from metabarcoding co-occur with changes in palynology and sea ice biomarkers, all demonstrating a shift from a permanent to seasonal sea ice regime. Furthermore, detailed bioinformatic analyses revealed previously unknown OTUs (cercozoans, dinoflagellates) in samples where traditional proxies indicate sea ice presence. Subsequently, the sea ice dinoflagellate *P. glacialis* was targeted with quantitative DNA techniques and traced in the geological record back to ~100,000 years ago, highlighting its potential as a sea ice tracer. But, in this study the link between the metabarcode data (OTUs) and sea ice in the geological record is demonstrated indirectly via other sea ice proxies. The relationship between metabarcode signatures, both on microbial eukaryotic community and individual protist species level, in marine sediments and sea ice environments needs to be established and calibrated in the modern environment to demonstrate the link between the sedimentary aDNA data and sea ice environments in the modern ocean. That will allow to fully develop sedimentary aDNA as an independent sea ice proxy and exploit its potential for understanding the evolution of the Arctic cryosphere. We demonstrate here that this approach has a tremendous and untapped potential, even in regions with oxygen-rich bottom waters [12].

### Data storage

Palynological, biomarker and ddPCR concentration data are freely available from the Bjerknes Centre Data Centre (BCDC) and www.pangaea.de at doi:10.1594/PANGAEA.900724. Metabarcoding sequence data is freely available from the public databases as a Sequence Read Archive with accession ID PRJEB27691.

## Supplementary information

Supplementary information

Supplementary Figure S1

Supplementary Figure S2

Supplementary Figure S3

Supplementary Table 4

Supplementary Table 1

Supplementary Table 2

Supplementary Table 3

Supplementary Captions
