# Supplementary material for: The potential of sedimentary ancient DNA for reconstructing past sea ice evolution
Source: ISME J. 2019 Jun 24;13(10):2566–77. doi: 10.1038/s41396-019-0457-1 (PMC6776040; doi:10.1038/s41396-019-0457-1)
Supplement: Supplementary file 1 — Supplementary information [file 41396_2019_457_MOESM1_ESM.docx]

**SUPPLEMENTARY INFORMATION TO:**

# **The potential of sedimentary ancient DNA for reconstructing past sea ice evolution**

Stijn De Schepper*^1^

Jessica L. Ray*^2^

Katrine Sandnes Skaar^2^

Henrik Sadatzki^3,7^

Umer Z. Ijaz^4^

Ruediger Stein^5,6^

Aud Larsen^2^

* These authors contributed equally to this work.

^1^ NORCE Climate, NORCE Norwegian Research Centre AS, Bjerknes Centre for Climate Research, Jahnebakken 5, 5007 Bergen, Norway; stde@norceresearch.no, +47 56107550.

^2^ NORCE Environment, NORCE Norwegian Research Centre AS, Nygårdsgaten 112, 5008 Bergen, Norway

^3^ Department of Earth Science, University of Bergen, Bjerknes Centre for Climate Research, Jahnebakken 5, 5007 Bergen, Norway.

^4^ University of Glasgow, School of Engineering, Oakfield Avenue, Glasgow G12 8LT, United Kingdom

^5^ Alfred Wegener Institute Helmholtz Centre for Polar and Marine Research, Am Alten Hafen 26, 27568 Bremerhaven, Germany

^6^ MARUM and Faculty of Geosciences, University of Bremen, P.O. Box 330440, 28334 Bremen, Germany

^7^ Present address: Research School of Earth Sciences, Australian National University, Canberra, ACT 2601, Australia

The supplementary information contains additional information on:

- Age model
- Sediment sampling routines for molecular analyses
- Metabarcoding laboratory protocols
- Bioinformatics
- Droplet digital PCR (ddPCR)
- Palynological laboratory procedures
- Biomarker laboratory procedures

## Age model

The age model for the Calypso core GS15-198-38CC is based on seven AMS ^14^C ages down to 345 cm, and a 5-cm resolution *N. pachyderma* sinistral isotope stratigraphy (1) below that level. For AMS ^14^C dating, we picked *N. pachyderma* sinistral from six samples of the Calypso core and from the sediment surface (0–1 cm) of the multicore. All AMS ^14^C dates were calibrated using Calib7.1 software (2), the Marine13 calibration curve (3) and no local reservoir age correction. The calibration was constrained on a 2σ range. The planktic stable oxygen isotope data (*N. pachyderma* sinistral) was correlated via three tie points to the global benthic stable oxygen isotope record of Lisiecki and Raymo (4). The final age model for the Calypso core is based on the ten age tie points listed below and linear interpolation between them.

| **Core** | **Depth in section** (cm) | **^14^C age** (BP) | **Error** (±) | **Two sigma** (cal yr BP) | | **Mean age** (cal yr BP) | **Lab** |
| --- | --- | --- | --- | --- | --- | --- | --- |
| 38MC-B | 1–2 | 410 | 15 | / | / | < 1903 | KECK |
| 38CC | 14–15 | 14 555 | 35 | 17 060 | 17 425 | 17 243 | KECK |
| 38CC | 60 | 15 170 | 50 | 17 793 | 18 139 | 17 966 | Beta |
| 38CC | 129–129.5 | 21 140 | 80 | 24 604 | 25 293 | 24 949 | KECK |
| 38CC | 180 | 22 720 | 110 | 26 209 | 27 004 | 26 607 | Beta |
| 38CC | 249–250 | 29 930 | 220 | 33 249 | 34 106 | 33 678 | KECK |
| 38CC | 345 | 42 150 | 560 | 44 126 | 46 130 | 45 128 | Beta |
|  | | | |  |  |  |  |
| **Core** | **Tie point depth in section**  (cm) | **Marine Isotope Stage transition** | **LR04 age**  **(**yr) |  |  |  |  |
| 38CC | 435 | MIS 3/4 | 57 000 |  |  |  |  |
| 38CC | 515 | MIS 4/5 | 71 000 |  |  |  |  |
| 38CC | 660 | MIS 5/5e | 123 000 |  |  |  |  |

MC = Multicore, CC = Calypso Core.

**Sediment sampling routines for molecular analyses in this study**

All frozen sediments for sedimentary ancient DNA (aDNA) analysis were handled in an access-restricted room intended for nucleic acid extraction, which is positively pressurized by a separate ventilation system employing HEPA filters. Laboratory work was conducted by personnel wearing single-use microfibre suits (3M™ Blue Disposable Protective Coveralls, 4515 Series), particle masks, safety goggles and gloves. The room and specifically-purposed equipment were thoroughly cleaned with a freshly-prepared 10% (v/v) solution of sodium hypochlorite in MQ water and DNAZap (Thermo Fisher Scientific) prior to use. All sample handling was conducted inside a class II laminar flow safety cabinet, whose internal surfaces were decontaminated using DNAZap (Invitrogen™ AM9890) and UV light prior to use. Sediment subsampling was conducted by two laboratory personnel of which one person worked always in the safety cabinet while the second person passed sediment samples to and received processed sediment samples from the first person. This was to ensure that the person in contact with the opened sediment samples worked exclusively inside the safety cabinet during subsampling.

**Metabarcoding laboratory protocols**

Samples in plastic bags were allowed to thaw at 4°C for 1-2 hours inside a purposed and pre-cleaned refrigerator in the sedimentary aDNA handling room. Syringes were removed from plastic bags inside a class II laminar flow safety cabinet and placed on a fresh strip of Parafilm M (Sigma Aldrich). The plunger was depressed in order to push the sediment core toward the bore end. The first 1 cm of exposed sediment was removed using a sterile scalpel and discarded. Six individual subsamples of approximately 0.5 g (wet weight) were weighed directly into DNeasy PowerBead tubes (QIAGEN PowerSoil kit, Hilden, Germany) and frozen at -20˚C until DNA purification. DNA purification was conducted using the DNeasy PowerSoil mini kit (QIAGEN) according to manufacturer instructions. All laboratory equipment and surfaces were cleaned again prior to every sample processing. DNA extraction was conducted in the same handling room where subsamples were taken. All pipetting steps during the DNA extraction protocol were conducted inside the same safety cabinet. Bead-assisted mechanical lysis was performed in a Tissue Lyzer II (Precellys, France) using the following program: 6000 rpm for 40 seconds, 2 min pause, 6000 rpm for 40 seconds, 2 min pause and 6000 rpm for 40 seconds. After purification, DNA was eluted in 100 µl kit elution buffer, divided into two aliquots of 50 µl and stored at -20˚C. One aliquot was maintained as archive, while the other aliquot was utilized as the working DNA stock and always handled inside a clean class II laminar flow safety cabinet.

To identify potential modern DNA contaminants in sedimentary aDNA preparations, we utilized both sampling and extraction controls. For sampling controls, PowerBead tubes (Axygen, Corning Life Sciences, New York, USA) were opened and placed in a tube rack inside the safety cabinet and left open for random time intervals during sediment subsampling (times were noted). These sampling controls were processed the same as sediment-containing samples for DNA extraction and PCR amplification. For extraction controls, PowerBead tubes containing no sediment material were run through the DNA extraction protocol exactly the same as sediment samples. Eluates from DNA extraction for both sampling and extraction controls were stored at -20˚C until PCR amplification.

We amplified the V7 hypervariable region of the small subunit ribosomal RNA (SSU rRNA) gene with universal eukaryote primers (5), to target a broad diversity of eukaryotic organisms. Preparation of metabarcoding libraries was conducted using a three-step PCR ligation library preparation protocol: initial amplification of target gene fragment using universal primers (Step 1), followed by Illumina MiSeq adapter ligation PCR using the same universal primers with Illumina adapter sequences (Step 2), and finally a dual-index barcode ligation PCR using primers specific for Illumina MiSeq adapter sequences (Step 3). All PCR master mixes (excluding template) were prepared in a restricted-access dedicated pre-PCR area inside a template-free laminar flow safety cabinet. Purified sedimentary aDNA templates (non-PCR-amplified) were then added to PCR reactions inside the class II laminar flow safety cabinet used for sediment subsampling and DNA extraction. Step 1 amplifications were performed in 25 µl PCR reactions containing 0.2 U Phusion High-Fidelity DNA polymerase (Thermo Fisher Scientific, Waltham, Massachusetts, USA), 1X HF buffer, 500 nmol of each primer F1183mod (5’-ATTTGACTCAACRCGGG-3’) and R1443mod (5’-GRGCATCACAGACCTG-3’) (ref. 5), 5 µmol of each dNTP, 5 µg molecular biology grade BSA (New England Biolabs, Ipswich, Massachusetts, USA), 5 µl template DNA and ultrapure water. The amplification program consisted of an initial denaturation at 95˚C for 5 min, 30 cycles of 95˚C for 20 s – 59˚C for 30 s – 72˚C for 30 s, and a final elongation at 72˚C for 10 min. PCR products were confirmed by agarose gel electrophoresis then twice-purified using HighPrep PCR magnetic beads (MagBio, Gaithersburg, Maryland, USA) at a PCR product:bead ratio of 1:1.8 for the first purification and 1.0 for the second purification. All manipulation of PCR products was conducted in dedicated post-PCR working areas with a separate ventilation system using purposed equipment and protective lab wear to ensure that no carry-over between pre- and post-PCR processes took place. The concentration of PCR products was measured using a Qubit fluorometer with High Sensitivity dsDNA assay kit (Thermo Fisher Scientific).

For step 2 PCR (Illumina MiSeq adapter ligation), 25 µl PCR reactions similar to the first round of amplification were prepared except that 10^7^ purified amplicons were used as template with primer N12seq-F1183mod (5’-ACACTCTTTCCCTACACGACGCTCTTCCGATCTNNNNNNNNNNNNAATTTGACTCAACRCGGG-3’) and primer N12seq-R1443mod (5’-GTGACTGGAGTTCAGACGTGTGCTCTTCCGATCTNNNNNNNNNNNNGRGCATCACAGACCTG). The same thermocycler program was used for the second round of amplification, with the exception that only 10 amplification cycles were run. PCR products were confirmed by agarose gel electrophoresis then twice-purified with magnetic beads (MagBio) at a PCR product:bead ratio of 1:1.8 for the first purification and 1:1 for the second purification. Purified PCR products were quantified using the Qubit HS dsDNA assay kit (Thermo Fisher Scientific).

For Step 3, the final PCR (Dual-index barcode ligation), 10^8^ purified amplicons were used as template in 50 µl PCR reactions containing unique forward-reverse Illumina barcode primer combinations P5D50XRead1seqp 5’-AATGATACGGCGACCACCGAGATCTACACNNNNNNNNACACTCTTTCCCTACACGACGCTCTTCCGATCT-3’

and

P7D70XRead2seqp 5’-CAAGCAGAAGACGGCATACGAGATNNNNNNNNGTGACTGGAGTTCAGACGTGTGCTCTTCCGATCT-3’ ,

where N_8_ stretches represent barcode sequences, for 15 cycles. PCR products were confirmed by agarose gel electrophoresis then purified with magnetic beads (MagBio) using a bead volume ratio of 1.8. After Qubit quantification, dual-indexed amplicon libraries were combined in equimolar ratios to yield a single pooled amplicon library. This library was purified with magnetic beads (MagBio) at a bead volume ratio of 1.0 to ensure thorough removal of residual primers and primer dimers, which was confirmed by agarose gel electrophoresis. The amplicon library was sequenced at the Norwegian Sequencing Centre (Oslo, Norway) on an Illumina MiSeq platform using v.3 PE300 chemistry. Demultiplexed sequence data were immediately archived on the Norwegian National Research Data Infrastructure (NIRD). Sequence data are publicly available through the European Nucleotide Archive (ENA) with Sequence Read Archive accession ID PRJEB27691.

## Bioinformatics

Raw fastq files were quality-trimmed using Sickle v.1.200 (6) with a 20 bp sliding window and Q20 cutoff with minimum length 10 bp. Error correction on paired-end reads was performed using BayesHammer (7) as implemented in the SPAdes assembler v.2.5.0 (8, 9). We utilized pandaseq (PAired-eND Assembler for DNA sequences (10)) to merge paired-end reads with a minimum 10 bp overlap. Recent work (11, 12) has shown that the above pipeline significantly reduces substitution rates (the primary type of error for Illumina MiSeq). Pooling, dereplication, sorting, singleton removal and OTU clustering at 97% similarity were performed using 64-bit VSEARCH v.2.8.1 for Linux x86 (13). VSEARCH was also utilized to perform *de novo* chimera removal (--uchime_denovo option), and to map reads back to OTUs. The assign_taxonomy.py script from the QIIME workflow (14) was used to taxonomically classify OTUs against the Protist Ribosomal Reference database (PR2) (15) v.4.10.0 using the *blast* algorithm (16) for best-hit identification. Phylogenetic distances between OTUs were resolved using kalign v2.0.4 (17) as a multisequence alignment (options –gpo 11 –gpe 0.85). Following this, FastTree v2.1.7 (18) generated the phylogenetic tree in NEWICK format and biome files for the OTUs were generated by combining the abundance table with taxonomy information using make_otu_table.py from the QIIME workflow. All statistical analyses were conducted in the R statistical computing environment (19).

**Droplet digital PCR**

Primers amplifying the ribosomal ITS1 region of *P.* *glacialis* were designed from an alignment of one *P. glacialis* database entry (Barcode of Life database DINO1190-08 from *P. glacialis* CCMP1383) with ITS1 sequences from other Suessiales dinoflagellates whose ITS1 sequences were publicly available in the BOLD database on 13 May 2018. Candidate primers *Polarella*-ITS-44F (5’-CGA CTG GGT GGA GAT GGT TG-3’) and *Polarella*-ITS-138R (5’-CCC AGG TGT TTA AGC CAG GT-3’) were subsequently tested for efficiency and specificity on a Bio-Rad QX200 droplet digital PCR machine (Bio-Rad, Carlsbad, California, USA) using EvaGreen Supermix with 250 nM final concentration of each primer. PCR reactions were performed in C1000 Touch thermocycler with deep-well module (Bio-Rad) using the following program: 95°C for 5 min, 40 cycles of 95°C for 30 sec, 62°C for 1 min, 4°C for 5 min, 90°C for 10 min and 4°C infinite hold. Primer specificity was confirmed by testing genomic DNA from cultures of *P. glacialias* RCC4283 (Roscoff Culture Collection, France), *Heterocapsa triquetra* K-1124 (NIVA, Oslo, Norway), *Gyrodinium aureolum* K-1555 (NIVA), *Karlodinium armiger* (ref. 20), *Woloszynska cincta* RCC2013, *Pelagodinium beii* RCC3593; *Alexandrium minutum* strains 76 and 77 (R. Siano, Ifremer, Brest, France), *Scripsiella donghaiensis* SC38 (R. Siano, Ifremer, Brest, France), *Symbiodinium* sp. RCC4011, and *Biechelaria* sp. RCC5518 (see figure below). PCR products from *P. glacialis* were then cloned using a standard cloning kit and Sanger sequenced to verify that all products had best match to ITS1 sequences from *P. glacialis* (data not shown). For quantitative amplification of *P. glacialis* ITS1 gene fragments from sedimentary aDNA, 20 µl ddPCR reactions containing 1X EvaGreen supermix, 250 nM of each primer and ultrapure water were prepared in a template-free pre-PCR area inside a laminar flow safety cabinet. Undiluted template DNA (5.5 µl) was always added to ddPCR reaction mix inside a class II laminar flow safety cabinet in a separate room. Droplet generation and post-PCR enumeration were performed according to manufacturer instructions (Bio-Rad). The ddPCR results were normalised to *P. glacialis* ITS1 copies g sediment^-1^. Raw data is available from www.pangaea.de.


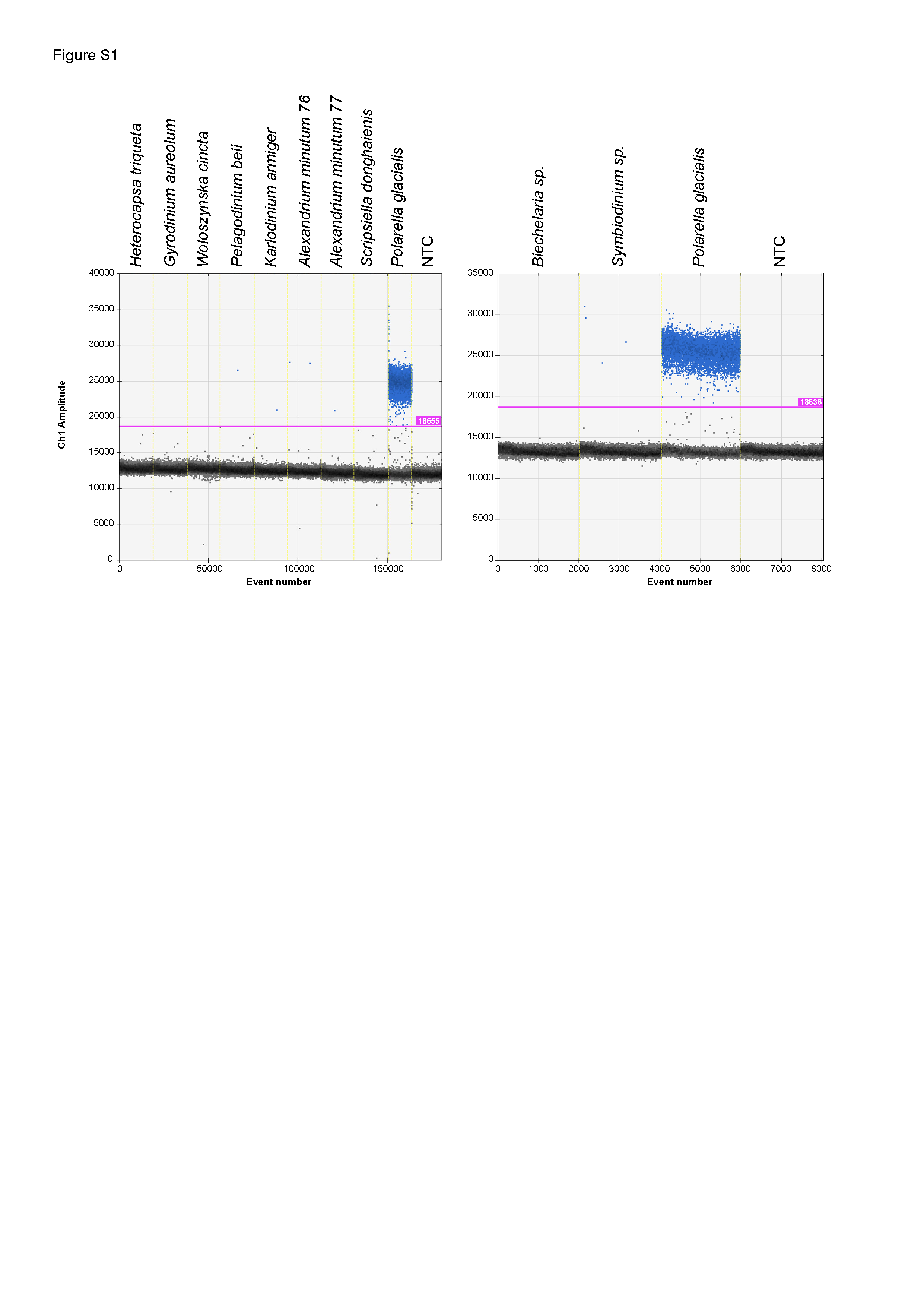


**Figure.** Scatterplot of ddPCR specificity test results for the *Polarella glacialis* ITS ddPCR assay. One hundred picograms of genomic DNA from each culture was used as template in 20 µl ddPCR reactions. The y-axis shows relative florescence intensity of detected droplets in each reaction. Cumulative number of droplets (“Events”) is indicated on the x-axis. At least 13,000 droplets were counted for all reactions. Blue points indicate “positive” droplets while grey/black points indicate “negative” droplets. Threshold for distinction between positive and negative droplets is indicated by the magenta trendline, which was manually adjusted to approximately 18600 RFU. Delayed arrival of *Biechelaria* sp. and *Symbiodinium* sp. cultures necessitated that these cultures were tested in a second set of reactions, and are therefore shown on a separate scatterplot.

## Palynology laboratory procedures

Between 1.7 and 13.2 grams of sediment were collected and prepared for palynological at Palynological Laboratory Services Ltd. (Holyhead, UK) using a standard procedure (e.g. De Schepper et al., 2017). This involved digesting the sediment in cold acids (HCl, HF), no oxidation or ultrasound. One *Lycopodium clavatum* spike (Batch 124961, n = 12542 ± 931 grains) was added prior to the acid digestion to estimate concentrations. Errors on concentrations were calculated according to (ref. 21). Organic residue was mounted on microscope slides, which were scanned along non-overlapping traverses using a 40x objective on a Zeiss Axio.Imager microscope. At least 10 traverses were completed, and when more than 10 specimens were counted within these traverses, the entire slide was scanned. Raw data is available from www.pangaea.de.

## Biomarker laboratory procedures

Total organic carbon and biomarker analyses were performed at the Alfred Wegener Institute (Bremerhaven, Germany) following a technique described in (22, 23). The total organic carbon content was measured on ~100 mg of freeze-dried and homogenized sediment using an ELTRA CS2000 Carbon Sulfur Determinator. Specific biomarkers were extracted from ~5 g freeze-dried and homogenized sediment using an accelerated solvent extractor (DIONEX, ASE200; 100°C, 5 min, 1,000 psi) and dichloromethane:methanol (2:1, v/v) as solvent, after adding internal standards to each sample. Biomarkers from the core-top sample were extracted by ultrasonication and using dichloromethane:methanol (2:1, v/v) as solvent (3 × 30 ml). Extracts were separated into hydrocarbon and sterol fractions by open-column chromatography and the sterol fraction was subsequently silylated with 200 µl BSTFA (bis-trimethylsilyl-trifluoroacet-amide) (60°C, 2 h). Biomarkers were analyzed by gas chromatography/mass spectrometry using an Agilent 7890B GC coupled to an Agilent 5977A mass selective detector (MSD) in selected ion monitoring mode for IP_25_, and an Agilent 6850 GC coupled with an Agilent 5975C VL MSD for sterols. IP_25_ was quantified using its molecular ion (m/z 350) in relation to the abundant fragment ion m/z 266 of the internal standard 7-HND. Dinosterol, brassicasterol, campesterol and ß-sitosterol were quantified as trimethylsilyl ethers using molecular ions m/z 500, m/z 470 m/z 472 and m/z 486 respectively, in relation to the molecular ion m/z 464 of the internal standard cholesterol-D_6_. Biomarker concentrations were normalized to the total organic carbon content.

Semi-quantitative sea ice estimates based on the phytoplankton-IP_25_ (PIP_25_) index were calculated following (ref. 24): PIP_25_ = IP_25_/(IP_25_ + phytoplankton biomarker * *c*). The PIP_25_ index was calculated using brassicasterol (P_B_IP_25_) or dinosterol (P_D_IP_25_) as phytoplankton biomarker. The balance factor *c* corresponds to the ratio of average IP_25_ and average phytoplankton biomarker concentration for GS15-198-38CC data presented here (c = 0.02 for P_B_IP_25_ and c = 0.18 for P_D_IP_25_). Raw data is available from www.pangaea.de.

**References**

1. Olsen IS. Investigations of abrupt climate change offshore east Greenland continental margin during Marine Isotope Stages 3 and 5 [MSc thesis]: University of Bergen; 2016.

2. Stuiver M, Reimer PJ, Reimer RW. CALIB 7.1 [WWW program] at <http://calib.org>. 2018.

3. Reimer PJ, Bard E, Bayliss A, Beck JW, Blackwell PG, Ramsey CB, et al. Intcal13 and Marine13 Radiocarbon Age Calibration Curves 0-50,000 Years Cal Bp. Radiocarbon. 2013;55(4):1869-87.

4. Lisiecki LE, Raymo M. A Pliocene-Pleistocene stack of 57 globally distributed benthic d18O records. Paleoceanography. 2005;20:PA1003.

5. Ray JL, Althammer J, Skaar KS, Simonelli P, Larsen A, Stoecker D, et al. Metabarcoding and metabolome analyses of copepod grazing reveal feeding preference and linkage to metabolite classes in dynamic microbial plankton communities. Mol Ecol. 2016;25(21):5585-602.

6. Joshi NA, Fass JN. Sickle: a Sliding-Window, Adaptive, Quality-Based Trimming Tool for FastQ Files (Version 1.33) [Software]. Available online at: <https://github.com/najoshi/sickle>. 2011.

7. Nikolenko SI, Korobeynikov AI, Alekseyev MA. BayesHammer: Bayesian clustering for error correction in single-cell sequencing. BMC Genomics. 2013;14.

8. Bankevich A, Nurk S, Antipov D, Gurevich AA, Dvorkin M, Kulikov AS, et al. SPAdes: A New Genome Assembly Algorithm and Its Applications to Single-Cell Sequencing. J Comput Biol. 2012;19(5):455-77.

9. Nurk S, Bankevich A, Antipov D, Gurevich AA, Korobeynikov A, Lapidus A, et al. Assembling Single-Cell Genomes and Mini-Metagenomes From Chimeric MDA Products. J Comput Biol. 2013;20(10):714-37.

10. Masella AP, Bartram AK, Truszkowski JM, Brown DG, Neufeld JD. PANDAseq: PAired-eND Assembler for Illumina sequences. BMC Bioinformatics. 2012;13.

11. Schirmer M, Ijaz UZ, D'Amore R, Hall N, Sloan WT, Quince C. Insight into biases and sequencing errors for amplicon sequencing with the Illumina MiSeq platform. Nucleic Acids Res. 2015;43(6).

12. D'Amore R, Ijaz UZ, Schirmer M, Kenny JG, Gregory R, Darby AC, et al. A comprehensive benchmarking study of protocols and sequencing platforms for 16S rRNA community profiling. BMC Genomics. 2016;17.

13. Rognes T, Flouri T, Nichols B, Quince C, Mahe F. VSEARCH: a versatile open source tool for metagenomics. PeerJ. 2016;4.

14. Caporaso JG, Kuczynski J, Stombaugh J, Bittinger K, Bushman FD, Costello EK, et al. QIIME allows analysis of high-throughput community sequencing data. Nat Methods. 2010;7(5):335-6.

15. Guillou L, Bachar D, Audic S, Bass D, Berney C, Bittner L, et al. The Protist Ribosomal Reference database (PR2): a catalog of unicellular eukaryote Small Sub-Unit rRNA sequences with curated taxonomy. Nucleic Acids Res. 2013;41(D1):D597-D604.

16. Altschul SF, Gish W, Miller W, Myers EW, Lipman DJ. Basic Local Alignment Search Tool. J Mol Biol. 1990;215(3):403-10.

17. Lassmann T, Sonnhammer ELL. Kalign - an accurate and fast multiple sequence alignment algorithm. BMC Bioinformatics. 2005;6.

18. Price MN, Dehal PS, Arkin AP. FastTree 2-Approximately Maximum-Likelihood Trees for Large Alignments. Plos One. 2010;5(3).

19. R Core Team. A language and environment for statistical computing. R Foundation for Statistical Computing, Vienna, Austria; 2018.

20. Berge T, Hansen PJ, Moestrup O. Prey size spectrum and bioenergetics of the mixotrophic dinoflagellate Karlodinium armiger. Aquat Microb Ecol. 2008;50(3):289-99.

21. Stockmarr J. Tablets with spores used in absolute pollen analysis. Pollen et Spores. 1971;13:615-21.

22. Fahl K, Stein R. Modern seasonal variability and deglacial/Holocene change of central Arctic Ocean sea-ice cover: New insights from biomarker proxy records. Earth Planet Sci Lett. 2012;351:123-33.

23. Sadatzki H, Dokken TM, Berben SMP, Muschitiello F, Stein R, Fahl K, et al. Sea ice variability in the southern Norwegian Sea during glacial Dansgaard–Oeschger climate cycles. Sci Adv. 2019;5(3):eaau6174.

24. Müller J, Wagner A, Fahl K, Stein R, Prange M, Lohmann G. Towards quantitative sea ice reconstructions in the northern North Atlantic: A combined biomarker and numerical modelling approach. Earth Planet Sci Lett. 2011;306(3-4):137-48.
