# Supplementary figures and images for: The potential of sedimentary ancient DNA for reconstructing past sea ice evolution
Source: ISME J. 2019 Jun 24;13(10):2566–77. doi: 10.1038/s41396-019-0457-1 (PMC6776040; doi:10.1038/s41396-019-0457-1)

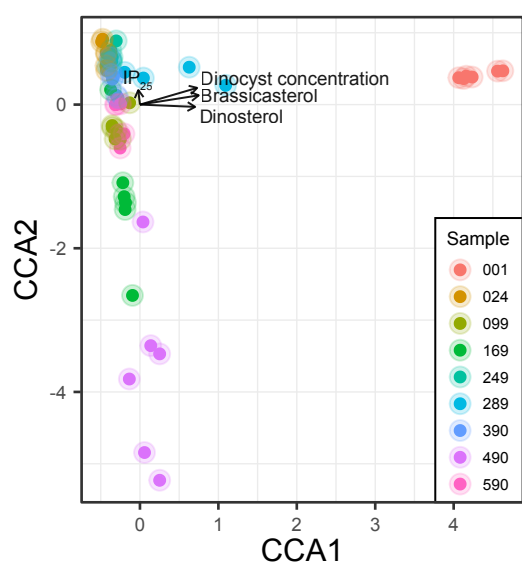

Supplement: Supplementary file 2 — Supplementary Figure S1 [file 41396_2019_457_MOESM2_ESM.pdf]

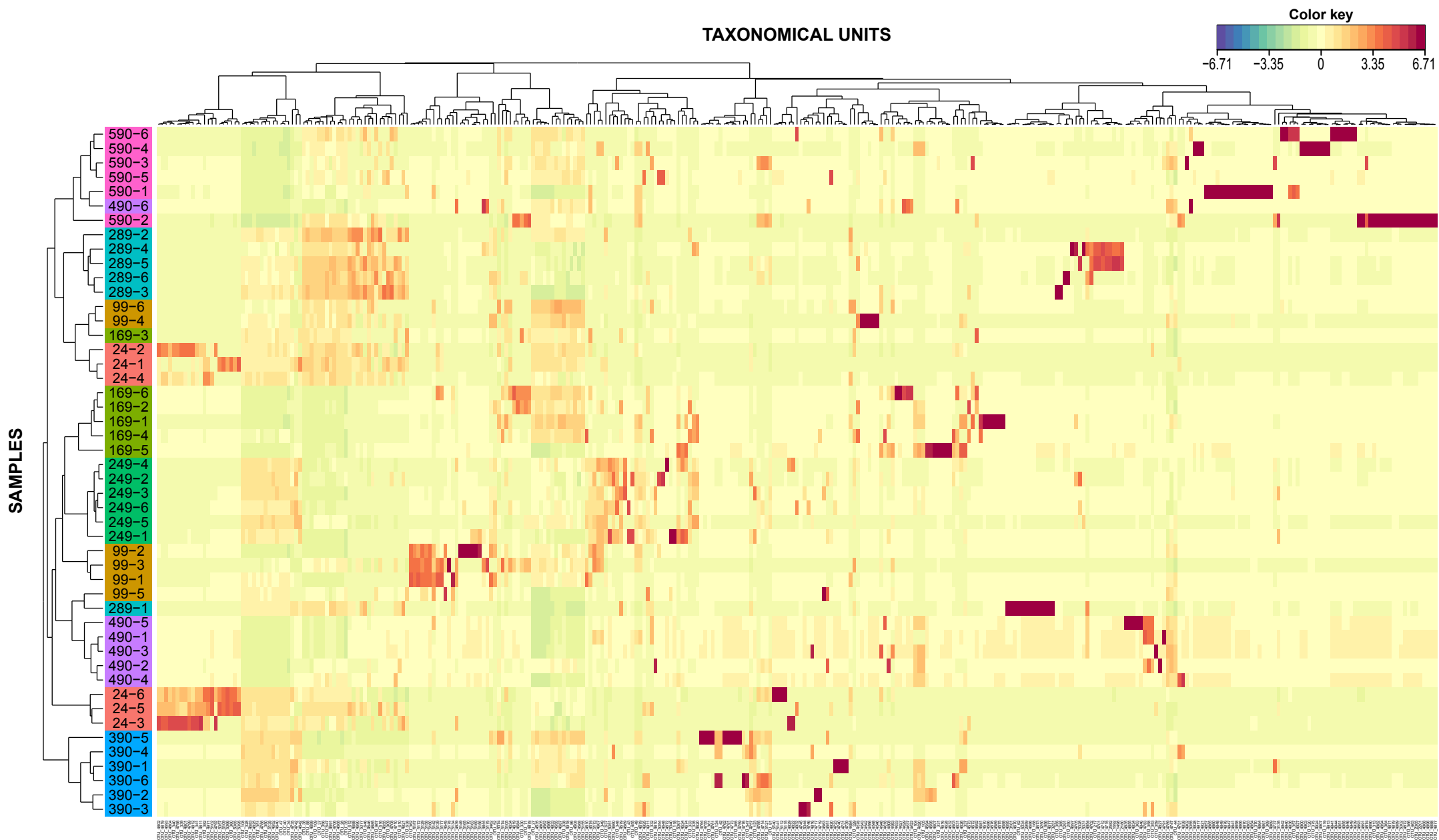

Supplement: Supplementary file 3 — Supplementary Figure S2 [file 41396_2019_457_MOESM3_ESM.pdf]
