# Supplementary material for: The potential of sedimentary ancient DNA for reconstructing past sea ice evolution
Source: ISME J. 2019 Jun 24;13(10):2566–77. doi: 10.1038/s41396-019-0457-1 (PMC6776040; doi:10.1038/s41396-019-0457-1)
Supplement: Supplementary file 9 — Supplementary Captions [file 41396_2019_457_MOESM9_ESM.docx]

**SUPPLEMENTARY FIGURES**

**Supplementary Figure S1.** Correspondence analysis of OTU diversity constrained by dinocyst concentration (cysts / g sediment), and dinosterol, brassicasterol and IP_25_ concentrations (µg/g TOC).

**Supplementary Figure S2.** Heatmap of OTUs with significant discriminatory power between different downcore samples as identified using sparse partial least squares discriminant analysis (sPLS-DA). Sample color as for Figure 3. Clustering on the y-axis indicates OTU-based community similarity between different samples, while clustering on the x-axis indicates agglomerative clustering based on OTU relative abundance counts.

**Supplementary Figure S3.** Heatmap showing results of correlation analysis between discriminant OTUs in downcore samples and dinoflagellate cyst abundances, brassicasterol, dinosterol and IP_25_ concentration. Color scale is continuous and indicates negative (blue) to positive (red) Kendall’s *tau* correlation coefficients. After correction for multiple comparisons according to (57), adjusted P-values are indicated with * for alpha = 0.05 and ** for alpha = 0.01.

**SUPPLEMENTARY TABLES**

**Supplementary Table 1.** Site GS15-198-38, sequence data processing metrics.

**Supplementary Table 2.** Alpha-diversity estimates of OTU results from metabarcoding of *sed*aDNA.

**Supplementary Table 3.** Site GS15-198-38, complete OTU table with PR2 taxonomic classifications.

**Supplementary Table 4**. Discriminant OTUs significantly correlated with environmental parameters. *P-values have been adjusted for multiple comparisons according to Benjamini and Hochberg (1995).
