# Supplementary material for: The potential of sedimentary ancient DNA for reconstructing past sea ice evolution
Source: ISME J. 2019 Jun 24;13(10):2566–77. doi: 10.1038/s41396-019-0457-1 (PMC6776040; doi:10.1038/s41396-019-0457-1)
Supplement: Supplementary file 4 — Supplementary Figure S3 [file 41396_2019_457_MOESM4_ESM.pdf]

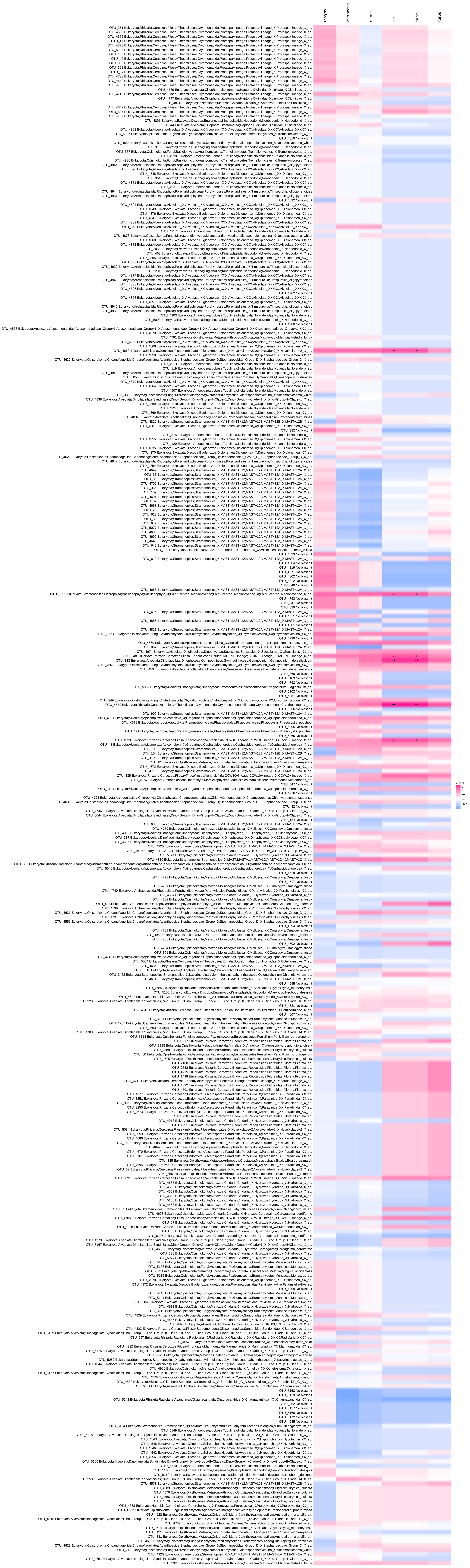
Dinocysts
Brassicasterol
Dinosterol
IP25
PBIP25
PDIP25
kendall
0.4
0.2
0.0
-0.2
